# *pmrCAB* Recombination Events among Colistin-Susceptible and -Resistant Acinetobacter baumannii Clinical Isolates Belonging to International Clone 7

**DOI:** 10.1128/msphere.00746-21

**Published:** 2021-12-01

**Authors:** Carolina Silva Nodari, Sebastian Alexander Fuchs, Kyriaki Xanthopoulou, Rodrigo Cayô, Harald Seifert, Ana Cristina Gales, Alexander Dilthey, Paul G. Higgins

**Affiliations:** a Universidade Federal de São Paulo-UNIFESP, Laboratório Alerta, Division of Infectious Diseases, Department of Internal Medicine, Escola Paulista de Medicina (EPM), São Paulo, Brazil; b Institute for Medical Microbiology, Immunology and Hygiene, Faculty of Medicine and University Hospital Cologne, University of Cologne, Cologne, Germany; c Institute of Medical Microbiology and Hospital Hygiene, Heinrich Heine University Düsseldorf, Düsseldorf, Germany; d German Center for Infection Research (DZIF), partner site Bonn-Cologne, Cologne, Germany; e Universidade Federal de São Paulo (UNIFESP), Laboratório de Bacteriologia e Imunologia (LIB), Setor de Biologia Molecular, Microbiologia e Imunologia, Departamento de Ciências Biológicas (DCB), Instituto de Ciências Ambientais, Químicas e Farmacêuticas (ICAQF), Diadema, Brazil; Antimicrobial Development Specialists, LLC

**Keywords:** polymyxins, colistin resistance, mobile genetic elements, insertion sequences, Gram-negative bacilli

## Abstract

Acinetobacter baumannii is a successful nosocomial pathogen due to its genomic plasticity. Homologous recombination allows genetic exchange and allelic variation among different clonal lineages and is one of the mechanisms associated with horizontal gene transfer (HGT) of resistance determinants. The main mechanism of colistin resistance in A. baumannii is mediated through mutations in the *pmrCAB* operon. Here, we describe two A. baumannii clinical isolates belonging to International Clone 7 (IC7) that have undergone recombination in the *pmrCAB* operon and evaluate the contribution of mobile genetic elements (MGE) to this phenomenon. Isolates 67569 and 72554 were colistin susceptible and resistant, respectively, and were submitted for short- and long-read genome sequencing using Illumina MiSeq and MinION platforms. Hybrid assemblies were built with Unicycler, and the assembled genomes were compared to reference genomes using NUCmer, Cortex, and SplitsTree. Genomes were annotated using Prokka, and MGEs were identified with ISfinder and repeat match. Both isolates presented a 21.5-kb recombining region encompassing *pmrCAB*. In isolate 67659, this region originated from IC5, while in isolate 72554 multiple recombination events might have happened, with the 5-kb recombining region encompassing *pmrCAB* associated with an isolate representing IC4. We could not identify MGEs involved in the mobilization of *pmrCAB* in these isolates. In summary, A. baumannii belonging to IC7 can present additional sequence divergence due to homologous recombination across clonal lineages. Such variation does not seem to be driven by antibiotic pressure but could contribute to HGT mediating colistin resistance.

**IMPORTANCE** Colistin resistance rates among Acinetobacter baumannii clinical isolates have increased over the last 20 years. Despite reports of the spread of plasmid-mediated colistin resistance among *Enterobacterales*, the presence of *mcr*-type genes in Acinetobacter spp. remains rare, and reduced colistin susceptibility is mainly associated with the acquisition of nonsynonymous mutations in *pmrCAB*. We have recently demonstrated that distinct *pmrCAB* sequences are associated with different A. baumannii International Clones (IC). In this study, we identified the presence of homologous recombination as an additional cause of genetic variation in this operon, which, to the best of our knowledge, was not mediated by mobile genetic elements. Even though this phenomenon was observed in both colistin-susceptible and -resistant isolates, it has the potential to contribute to the spread of resistance-conferring alleles, leading to reduced susceptibility to this last-resort antimicrobial agent.

## INTRODUCTION

Acinetobacter baumannii is an opportunistic pathogen causing a variety of difficult-to-treat infections owing to their high incidence of antimicrobial resistance. One of the reasons for this is its high genomic plasticity and its ability to acquire resistance determinants (1, 2). The A. baumannii population can be grouped into nine international clonal lineages (3), which differ from each other in at least 1,800 alleles, as shown by core genome multilocus sequence typing (cgMLST) (4). Furthermore, each lineage has distinct alleles associated with them, such as the intrinsic *bla*_OXA-51_-like (5).

Homologous recombination allows foreign DNA to be integrated into the chromosome, and in A. baumannii it has already been associated with the acquisition of resistance determinants to aminoglycosides (6, 7). Additionally, other studies have shown that homologous recombination contributes to the allelic variation of intrinsic resistance determinants, such as the outer membrane protein CarO (8) and the chromosome-encoded Acinetobacter-derived cephalosporinase (ADC) (9).

Mutations in the *pmrCAB* operon are the main mechanism causing reduced susceptibility to colistin among A. baumannii strains (10). We have recently demonstrated the allelic variation of *pmrCAB* between distinct International Clones (ICs) and that colistin-susceptible isolates belonging to the same clonal lineage should be used as reference strains when investigating point mutations potentially associated with colistin resistance (11, 12). Interestingly, some of the IC2 isolates described in the study by Gerson and colleagues (11) presented *pmrCAB* sequences that are associated with IC4, suggesting homologous recombination between these clonal lineages. Kim and Ko (13) have also suggested that *pmrCAB* genetic variation between distinct species belonging to the A. baumannii-A. calcoaceticus complex was due to recombination.

Here, we describe two A. baumannii clinical isolates belonging to IC7 with distinct colistin susceptibility profiles and presenting recombined *pmrCAB* operons and evaluate the contribution of mobile genetic elements (MGE) to this phenomenon.

(This work was presented in part at the 12th International Symposium on the Biology of Acinetobacter in Frankfurt, Germany, 2019)

## RESULTS AND DISCUSSION

**FIG 1:**
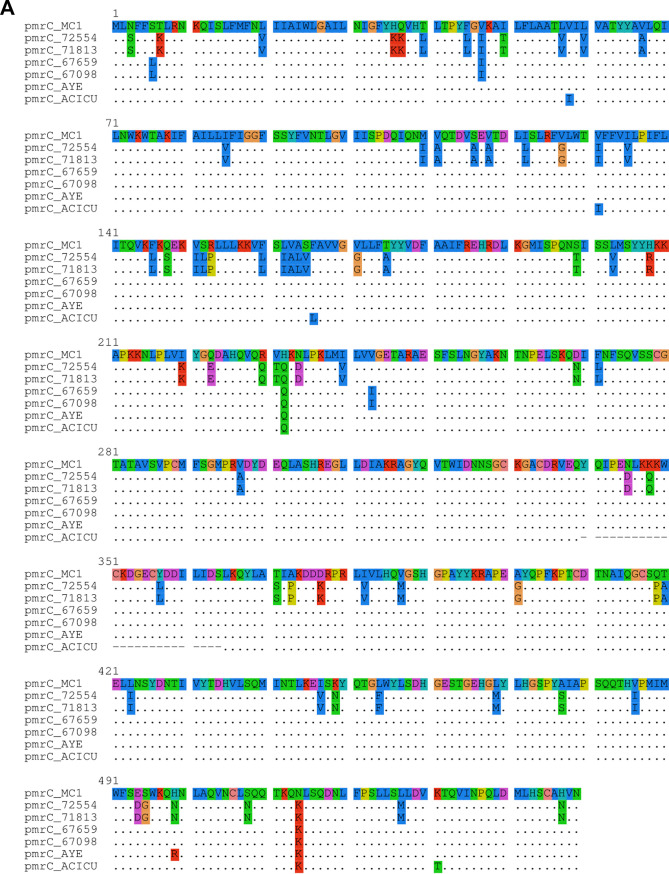

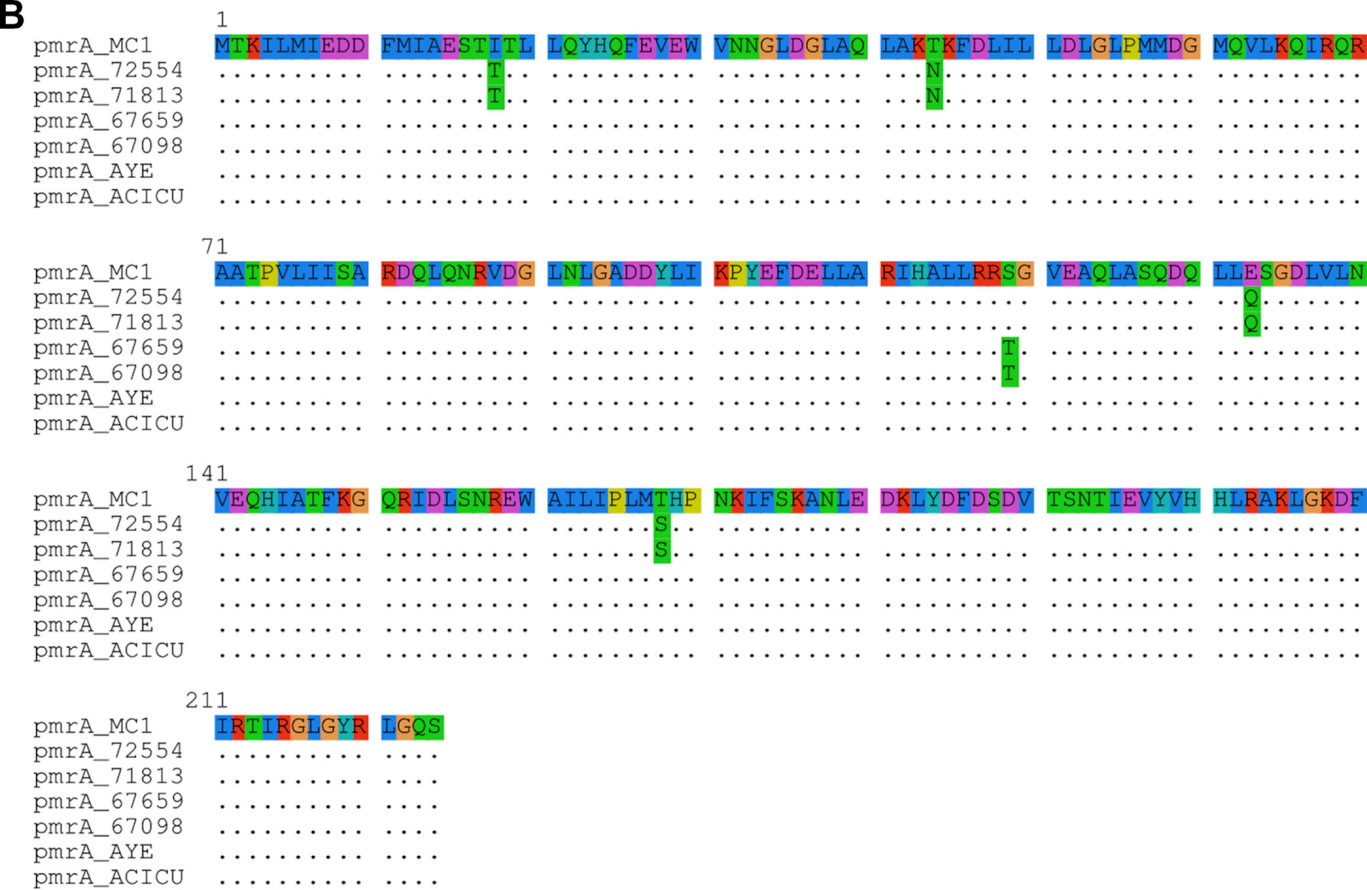

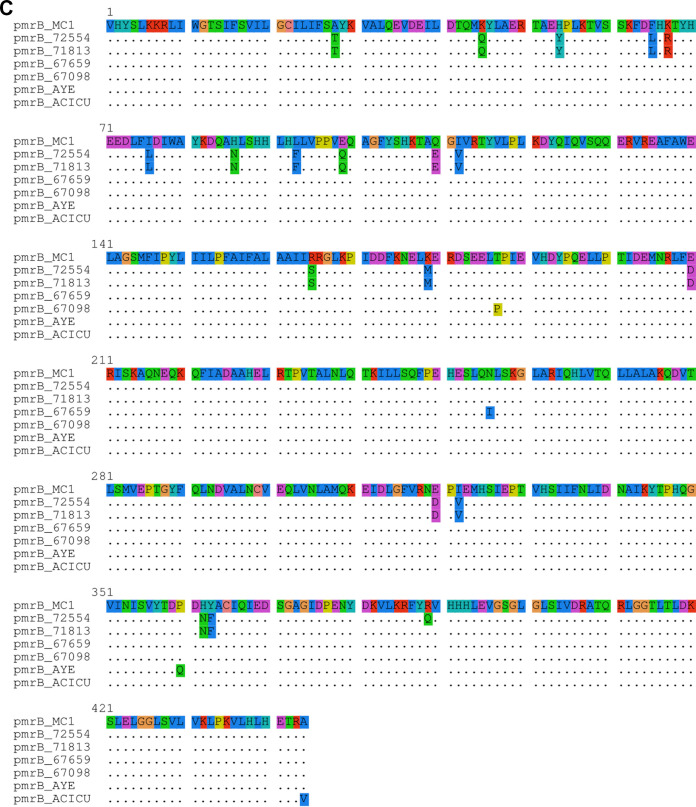

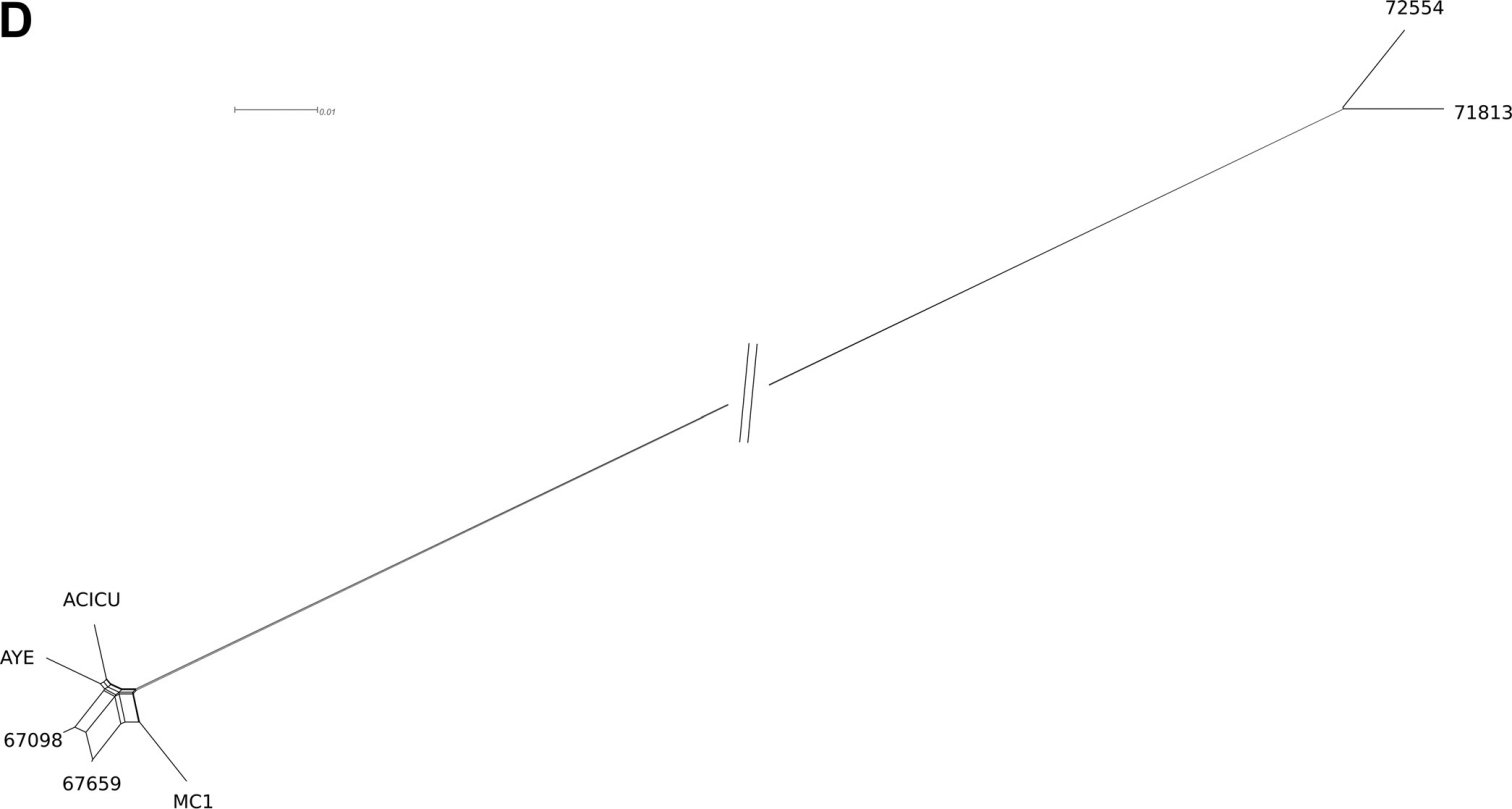
(A to D) Protein sequence alignment of PmrC (A), PmrA (B), and PmrB (C) and SplitsTree-based neighbor-net of a 23.6-kb genomic region encompassing *pmrCAB* (D) between isolates MC1 (IC7), 72554 (IC7), 71813 (IC4), 67659 (IC7), 67098 (IC5), AYE (IC1), and ACICU (IC2). Sequences belonging to isolate MC1 were used as references for sequence alignment. Amino acid differences are highlighted in colors (panels A to C).

**FIG 2:**
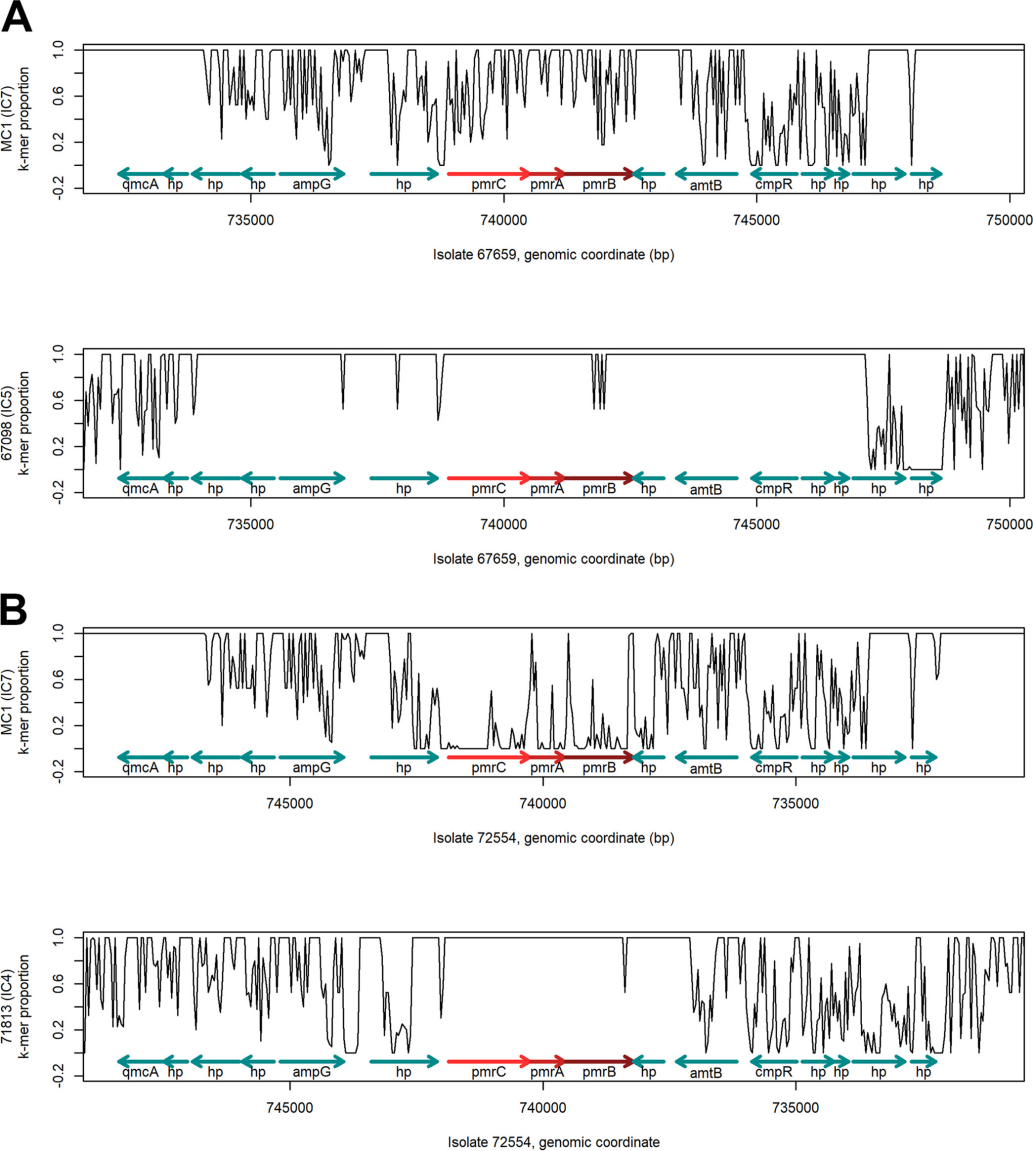
(A and B) Spatial k-mer sharing plots of a 23.6-kb genomic region encompassing *pmrCAB* and flanking genes of isolate 67659 against isolates MC1 (IC7, top) and 67098 (IC5, bottom) (A) and 72554 against MC1 (IC7, top) and 71813 (IC4, bottom) (B). The plots show spatial variations in the proportion of k-mers present in the genomes described on the *x* axis also present in the genome of the different references described on the *y* axis, calculated in sliding windows of 40 bases along the genome of the first isolate and for *k *= 19. Plots are based on k-mer counts computed with Cortex and a custom R visualization script. *pmrCAB* coding regions are highlighted in red, and flanking genes are indicated in green.

Some divergence was observed when the PmrCAB protein sequences of the IC7 isolates 67659 and 72554 were aligned against MC1 (IC7 reference genome). The colistin-susceptible isolate 67659 showed one amino acid substitution in both PmrA and PmrB as well as five in PmrC. In contrast, isolate 72554 presented 4, 18, and 71 amino acid substitutions in PmrA, PmrB, and PmrC, respectively (Fig. 1A to C). The k-mer sharing analysis of *pmrCAB* and its flanking regions demonstrated that sequence similarities were increased when isolates 67659 and 72554 were compared to those belonging to IC5 and IC4, respectively (Fig. 2). Furthermore, no amino acid substitutions were observed in PmrC or PmrA when isolates 67659 and 72554 were compared against isolate 67098 (IC5) and isolate 71813 (IC4), respectively. Higher sequence similarity was also observed in PmrB, with only a single substitution (Arg_389_Gln) identified when isolates 71813 and 72554 were compared, as well as two substitutions (Pro_187_Thr and Asn_256_Ile) in the comparison between isolates 67098 and 67659 (Fig. 1A to C). The representativeness of the included reference genomes was also explored in an additional set of isolates as well as in a larger genomic region (see Fig. S1 to S5 in the supplemental material).

10.1128/msphere.00746-21.2 FIG S1 Spatial k-mer sharing plots of *pmrCAB* and flanking regions of isolates belonging to IC7 against MC1. The plots show spatial variations in the proportion of k-mers present in MC1 also observed in the genome of the different isolates described in the *y* axis, calculated in sliding windows of 40 bases along the genome of the MC1 and for *k* = 19. Plots are based on k-mer counts computed with Cortex and a custom R visualization script. *pmrCAB* coding regions are highlighted in red, and flanking genes are indicated in green. Download FIG S1, PDF file, 0.9 MB. Copyright © 2021 Nodari et al. 2021 Nodari et al. https://creativecommons.org/licenses/by/4.0/ This content is distributed under the terms of the Creative Commons Attribution 4.0 International license.

10.1128/msphere.00746-21.3 FIG S2 Spatial k-mer sharing plots of *pmrCAB* and flanking regions of isolates belonging to IC4 against 71813. The plots show spatial variations in the proportion of k-mers present in 71813 also observed in the genome of the different isolates described on the *y* axis, calculated in sliding windows of 40 bases along the genome of the 71813 and for *k* = 19. Plots are based on k-mer counts computed with Cortex and a custom R visualization script. *pmrCAB* coding regions are highlighted in red, and flanking genes are indicated in green. Download FIG S2, PDF file, 0.5 MB. Copyright © 2021 Nodari et al. 2021 Nodari et al. https://creativecommons.org/licenses/by/4.0/ This content is distributed under the terms of the Creative Commons Attribution 4.0 International license.

10.1128/msphere.00746-21.4 FIG S3 Spatial k-mer sharing plots of *pmrCAB* and flanking regions of isolates belonging to IC5 against 67098. The plots show spatial variations in the proportion of k-mers present in 67098 also observed in the genome of the different isolates described on the *y* axis, calculated in sliding windows of 40 bases along the genome of the 67098 and for *k* = 19. Plots are based on k-mer counts computed with Cortex and a custom R visualization script. *pmrCAB* coding regions are highlighted in red, and flanking genes are indicated in green. Download FIG S3, PDF file, 0.9 MB. Copyright © 2021 Nodari et al. 2021 Nodari et al. https://creativecommons.org/licenses/by/4.0/ This content is distributed under the terms of the Creative Commons Attribution 4.0 International license.

10.1128/msphere.00746-21.5 FIG S4 Spatial k-mer sharing plots of a 500-kb genomic region encompassing *pmrCAB* and flanking genes of isolate 67659 against isolates MC1 (IC7, top) and 67098 (IC5, bottom). The plots show spatial variations in the proportion of k-mers present in the genomes described on the *x* axis also present in the genome of the different references described on the *y* axis, calculated in sliding windows of 40 bases along the genome of the first isolate and for *k* = 19. Plots are based on k-mer counts computed with Cortex and a custom R visualization script. *pmrCAB* coding regions are highlighted in red. The shaded grey area highlights the genomic region depicted in Fig. 1A. Download FIG S4, PDF file, 0.1 MB. Copyright © 2021 Nodari et al. 2021 Nodari et al. https://creativecommons.org/licenses/by/4.0/ This content is distributed under the terms of the Creative Commons Attribution 4.0 International license.

10.1128/msphere.00746-21.6 FIG S5 Spatial k-mer sharing plots of a 500-kb genomic region encompassing *pmrCAB* and flanking genes of isolate 72554 against isolates MC1 (IC7, top) and 71813 (IC4, bottom). The plots show spatial variations in the proportion of k-mers present in the genomes described on the *x* axis also present in the genome of the different references described in the *y* axis, calculated in sliding windows of 40 bases along the genome of the first isolate and for *k* = 19. Plots are based on k-mer counts computed with Cortex and a custom R visualization script. *pmrCAB* coding regions are highlighted in red. The shaded grey areas highlight the genomic region depicted in Fig. 1B. Download FIG S5, PDF file, 0.08 MB. Copyright © 2021 Nodari et al. 2021 Nodari et al. https://creativecommons.org/licenses/by/4.0/ This content is distributed under the terms of the Creative Commons Attribution 4.0 International license.

The presence of regions with such high polymorphism rates suggests that horizontal transfer through recombination, rather than the accumulation of multiple point mutations over time, is involved in the variability of these specific DNA fragments. This is particularly important and more frequent in naturally transformable species, such as A. baumannii (1, 2). Based on the large number of nonsynonymous mutations observed in *pmrCAB*, with PmrC protein sequences presenting up to 13% divergence from what is expected for their lineage, we can infer that this operon has been transferred across clonal lineages through homologous recombination. The likely presence of recombination around the *pmrCAB* operon was confirmed by a SplitsTree analysis, also including reference genomes for IC1 and IC2 (Fig. 1D; phi test for recombination, *P* = 0.0). Considering that IC4 and IC5, together with IC7, are the most frequent lineages observed in South America (3) and were already described in the same hospital (12, 14), it comes as no surprise that horizontal gene transfer occurred among those lineages.

Using a k-mer-based analysis, it was noticed that the length of the region presenting high sequence divergence surrounding *pmrCAB* was similar between the two evaluated isolates and extended to at least 8 kb up- and downstream of *pmrCAB* (Fig. 2A and B, top). However, when using the same approach to compare those isolates to the reference genomes belonging to IC4 and IC5, which presumably acted as donors of the recombining regions, some differences were observed. While k-mer sharing proportion between isolates 67659 and 67098 was close to 1 through the whole extension of the recombining region (Fig. 2A, bottom), the similarities between isolates 72554 and 71813 were restricted to only 700 bp upstream of *pmrC* as well as 1,000 bp downstream of *pmrB* (Fig. 2B, bottom). This finding suggests that additional recombination events have taken place and that the *pmrCAB* allele belonging to IC4 went through some other intermediary host before making it into 72554, consistent with SplitsTree results. Boinett and colleagues (15) have previously suggested that a 700-kb genomic region that included *pmrCAB* had undergone homologous recombination in laboratory-induced colistin-resistant isolates. Those isolates, however, belonged to IC2, suggesting that recombining regions vary depending on their genetic background. This observation would be in agreement to the phenomenon described by Kim and Ko (13), where the authors reported that recombination could happen within *pmrC*, generating mosaic alleles. Such variation, however, was not observed in either of the two isolates evaluated in this study.

MGEs are often involved in horizontal gene transfer and, in A. baumannii, are frequently related to insertion sequences (ISs) and/or composite transposons (7, 16). Despite multiple copies of distinct IS elements being identified in the genomes of isolates 67659 and 72554 (data not shown), none of them was observed within or flanking the recombining region encompassing *pmrCAB*. In fact, the nearest IS detected was a copy of IS*Aba125* that was ∼14 kb upstream of *pmrC* in both isolates, while in the other direction the closest IS element identified (a copy of IS*17*) was located >120 kb downstream of *pmrB*, suggesting that recombination was not mediated by DNA mobilization either through an IS or a composite transposon. Phage-related structures were also observed through the genome of both isolates. However, similar to the IS elements, none of them was found flanking the recombining regions, and the closest intact phage was observed >300 kb downstream of *pmrB*.

Considering that IS elements are self-transposable structures (17), we investigated the presence of inverted repeats flanking the recombining region, since they indicate that MGEs were lost postrecombination. A large number of repeats was observed within and flanking the recombining region in both isolates, with an average of 44 repeats per 1,000 bp. However, sequence analysis revealed that none of them were part of or constituted an insertion site for known IS elements. Moreover, they were also found at the same position in isolates 67098 and 71813, suggesting that they were translocated from IC5 and IC4 to IC7 during recombination, respectively, rather than being responsible for the DNA mobilization. Therefore, the mechanisms involved in the mobilization of *pmrCAB* into IC7 isolates remain to be elucidated.

Allelic variation in the *pmrCAB* operon is associated with natural polymorphisms within each A. baumannii IC. In our study, we demonstrated that IC7 isolates can present additional sequence divergence as a consequence of homologous recombination of regions with variable lengths across distinct clonal lineages. Interestingly, the recombination appears not to be driven by antibiotic pressure, since it was observed in both colistin-susceptible and -resistant isolates, and a variety of clonal lineages can act as donors of the recombining region. Additionally, we observed that MGEs were not required for the transfer of *pmrCAB* in our isolates, since neither IS elements nor other MGEs were detected flanking the recombining region. Further studies are required to determine the mechanisms driving the mobilization of *pmrCAB* and to evaluate the presence of this phenomenon in other ICs as well as its frequency in the A. baumannii population.

## MATERIALS AND METHODS

### Bacterial isolates.

A. baumannii clinical isolates 67659 and 72554 were recovered from the same tertiary hospital in the city of São Paulo, Brazil, 2 years apart (2015 and 2017, respectively). Their antimicrobial susceptibility profile was previously determined (14), and they were found to be colistin susceptible (MIC, 1 mg/liter) and resistant (MIC, >128 mg/liter), respectively. Their genomes were previously sequenced using the Illumina MiSeq platform, and cgMLST analysis revealed that the isolates had 28 allele differences and were grouped under IC7 (14). Additionally, previously described colistin-susceptible isolates belonging to IC4 (71813), IC5 (67098), and IC7 (MC1) were included as reference genomes for each IC (14, 18).

### Long-read WGS using MinION platform.

Genomic DNA of isolates 67659 and 72554 was extracted using the Genomic-Tips 100/G kit and genomic DNA buffers kit (Qiagen, Hilden, Germany). Libraries were prepared using the ligation sequencing kit (SQK-LSK109), combined with a native barcoding kit (EXP-NBD104) and the rapid barcoding kit (SQK-RBK004) (Oxford Nanopore Technologies, Oxford, United Kingdom), and were loaded onto an R9.4 flow cell (Oxford Nanopore Technologies). Genomes were assembled with a hybrid approach using Unicycler version 0.4.4 (19) with default parameters.

### Genome alignment and identification of the recombining region including *pmrCAB*.

The exact position of the *pmrCAB* operon was identified by aligning the *pmrCAB* sequence from A. baumannii ATCC 19606 (GenBank accession number NZ_CP045110.1) against the hybrid assemblies using the NUCmer tool of the MUMmer package, version 4.0.0beta2 (20), with default parameters. K-mer sharing plots were used for the robust identification of sequence homologies and recombination boundaries between lineages by visualizing spatial variation in the proportion of k-mers from one isolate (X) also present in another isolate (Y), calculated in sliding windows of 40 bases along the genome of X. In contrast to other alignment approaches, k-mer sharing plots do not require full assembly of genome Y but can be created based on short-read-derived k-mer counts. For a given region in isolate X, k-mer sharing values close to 1 indicate the likely presence of a homologous region in Y, whereas lower values indicate reduced similarity or the absence of the corresponding region from Y. The k-mer sharing plots were used to determine sequence homology patterns between different isolates around the *pmrCAB* operon and were created with a custom R script executed in RStudio (version 1.3.1093) (21). k-mer presence or absence was determined with Cortex (version 1.0.5.21; options “–mem_height 25,” “–mem_width 100,” and “–kmer_size 19”) (22), employing a minimum k-mer coverage threshold of 10 for the analysis of short-read data. A neighbor-net analysis of the *pmrCAB* region was carried out with SplitsTree (23) with default settings, based on a MUSCLE (24) multiple-sequence alignment of identified *pmrCAB* sequences plus 10 kb of adjacent sequence from either side of *pmrCAB*. The phi test implemented in SplitsTree (null hypothesis: no recombination) was used to test for recombination.

### Characterization of the mobile structures involved in *pmrCAB* recombination.

To fully annotate the hybrid assemblies and to search for MGEs, Prokka version 1.14.5 (25) was used with default parameters. Putative IS elements and phage-related structures were further identified with the blast tools of IS-finder (https://isfinder.biotoul.fr/) and Phaster (https://phaster.ca/), respectively, using default parameters. Inverted repeats (IR) were identified using the repeat-match tool of the MUMmer package version 4.0.0beta2 (20) with a minimum repeat length of 10 bases.

### Data availability.

Short and long raw reads generated for IC7 isolates 67659 and 72554, as well as the reference isolates 67098 and 71813, were submitted to the Sequence Read Archive (https://www.ncbi.nlm.nih.gov/sra/) of the National Center for Biotechnology Information (NCBI) under BioProject number PRJNA632943. Genome data from isolate MC1 are available under GenBank accession number NZ_QXPV00000000.1. Additional isolates presented in the supplemental material had their short raw reads submitted to the European Nucleotide Archive (http://www.ebi.ac.uk/ena/) of EMBL European Bioinformatics Institute (EBI) under the study accession numbers PRJEB12082 and PRJEB27899.

10.1128/msphere.00746-21.1 TABLE S1 Genome assembly statistics and inferred location of *pmrCAB* in A. baumannii clinical isolates included in the study. Download Table S1, PDF file, 0.02 MB. Copyright © 2021 Nodari et al. 2021 Nodari et al. https://creativecommons.org/licenses/by/4.0/ This content is distributed under the terms of the Creative Commons Attribution 4.0 International license.
