# Supplementary figures and images for: pmrCAB Recombination Events among Colistin-Susceptible and -Resistant Acinetobacter baumannii Clinical Isolates Belonging to International Clone 7
Source: mSphere. 2021 Dec 1;6(6):e00746-21. doi: 10.1128/msphere.00746-21 (PMC8636104; doi:10.1128/msphere.00746-21)

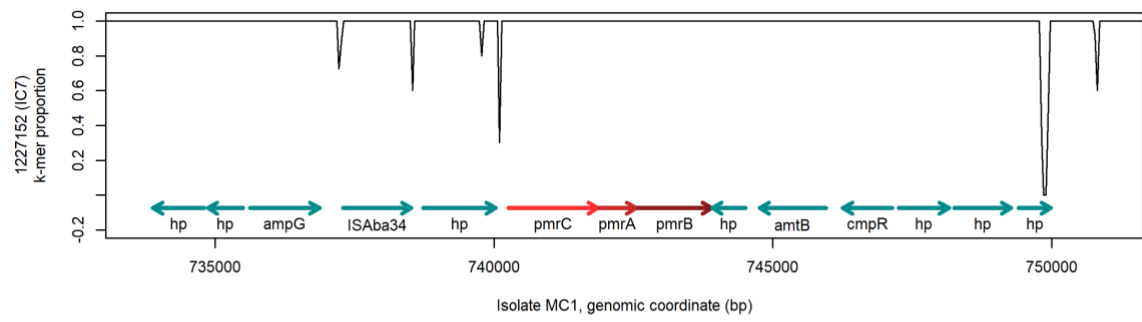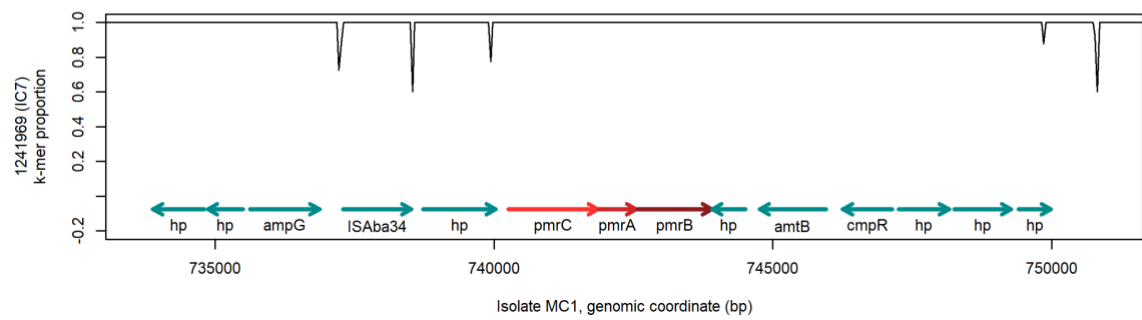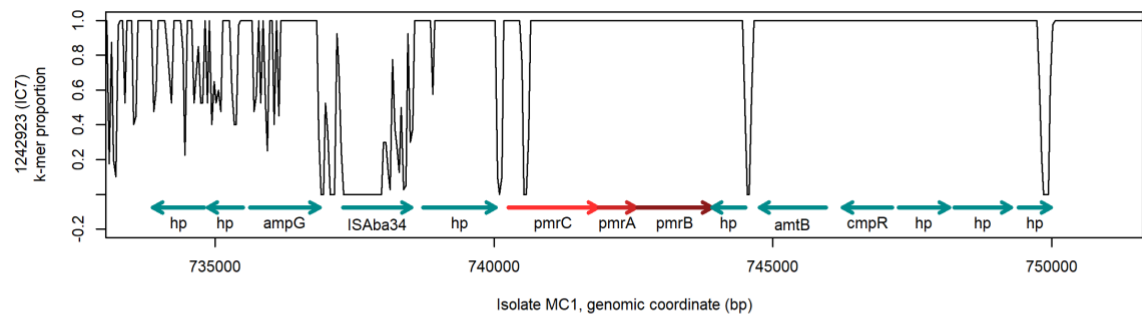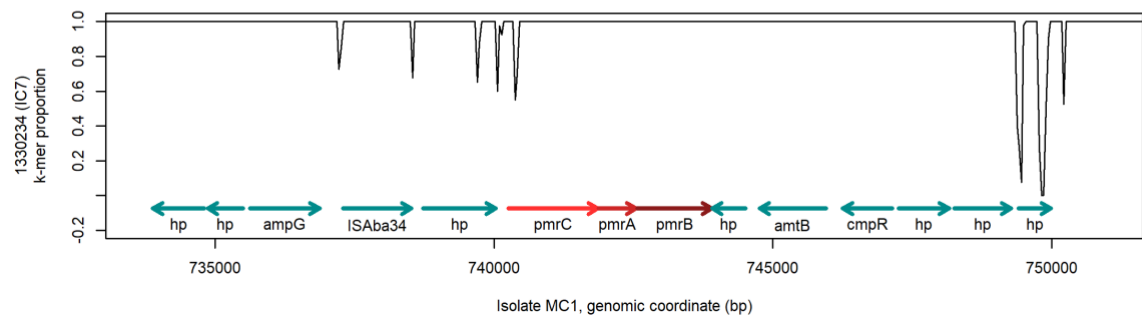

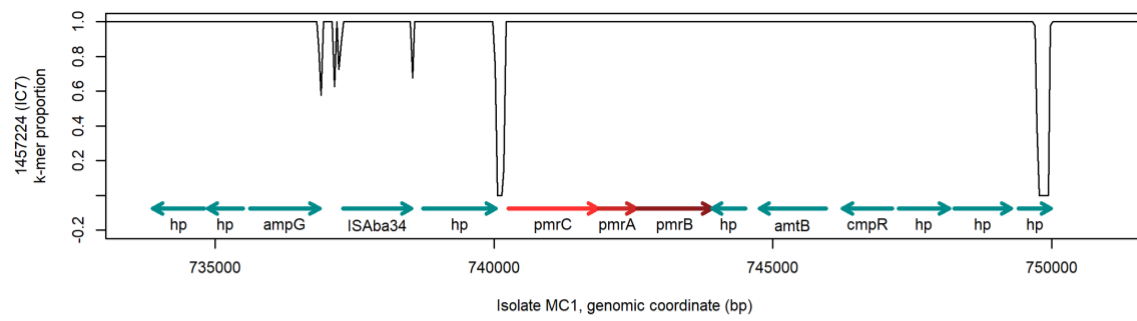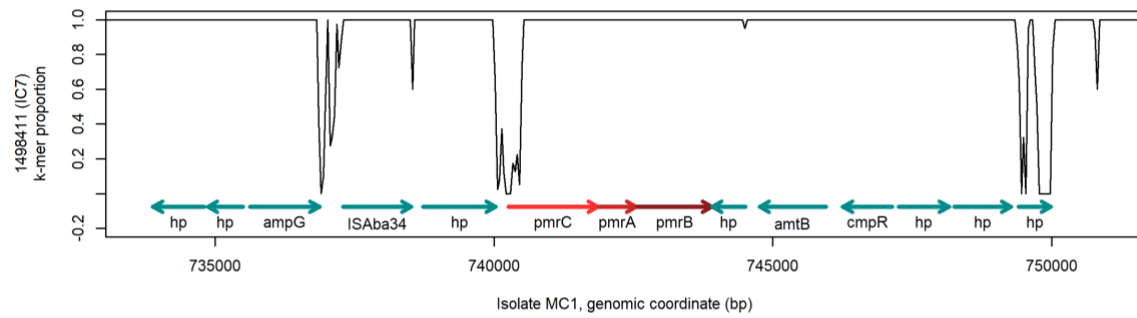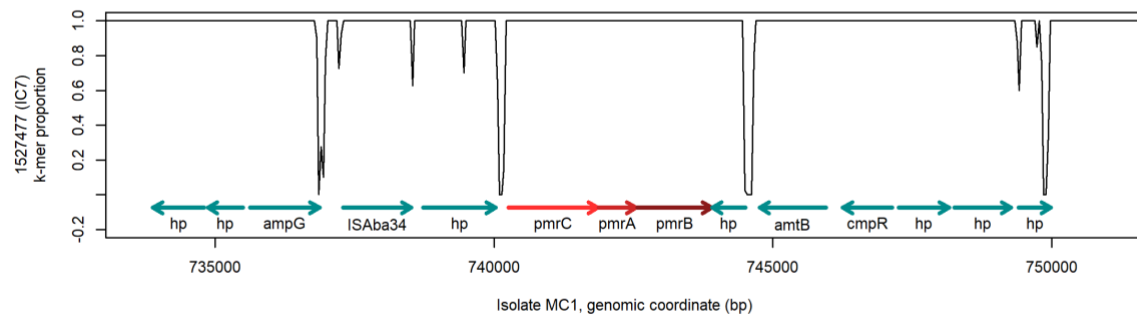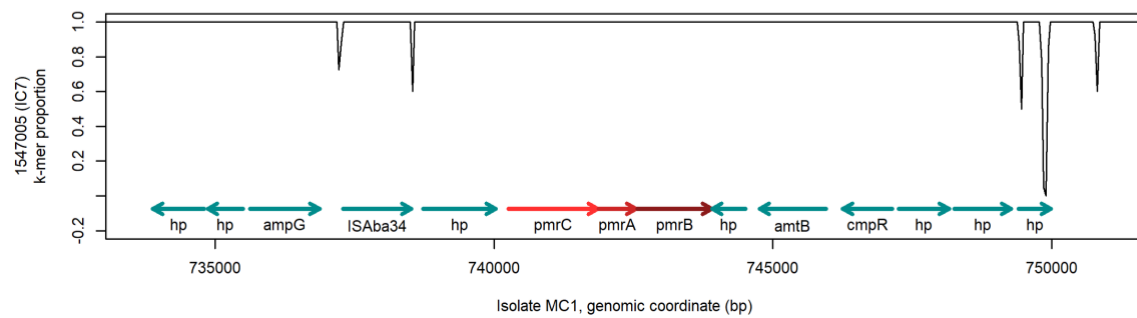

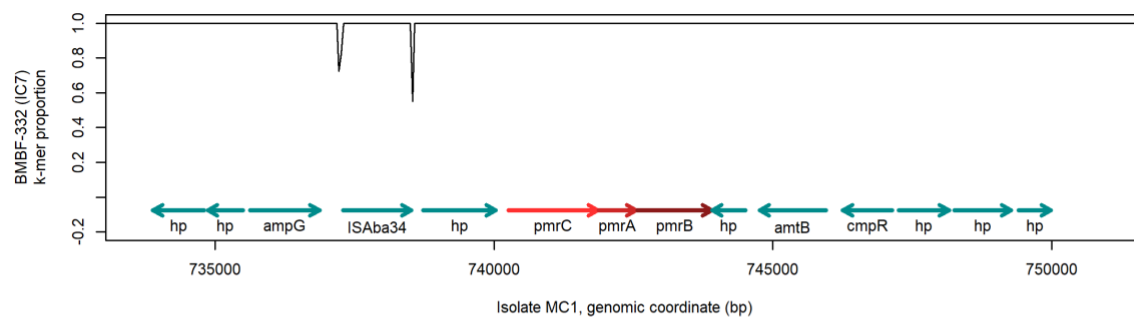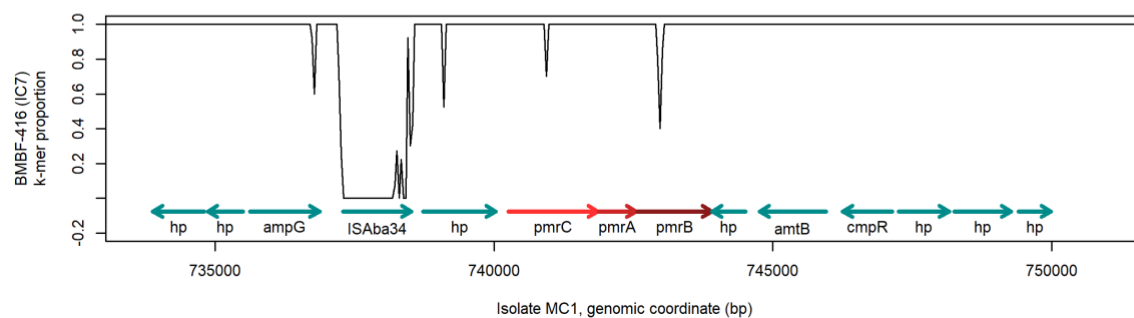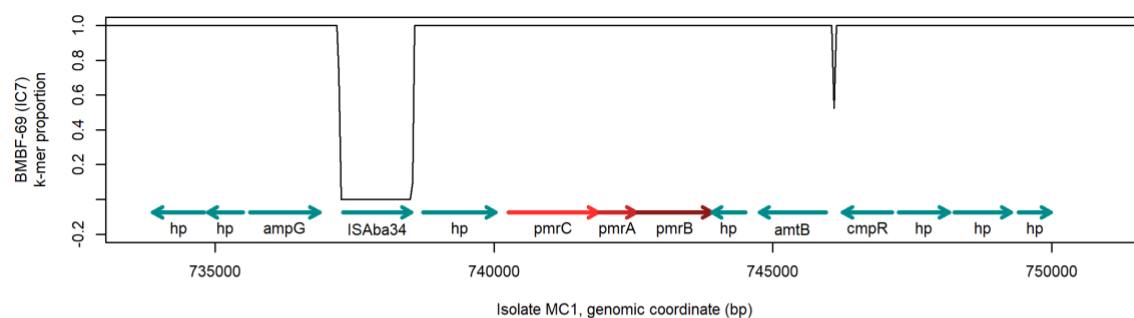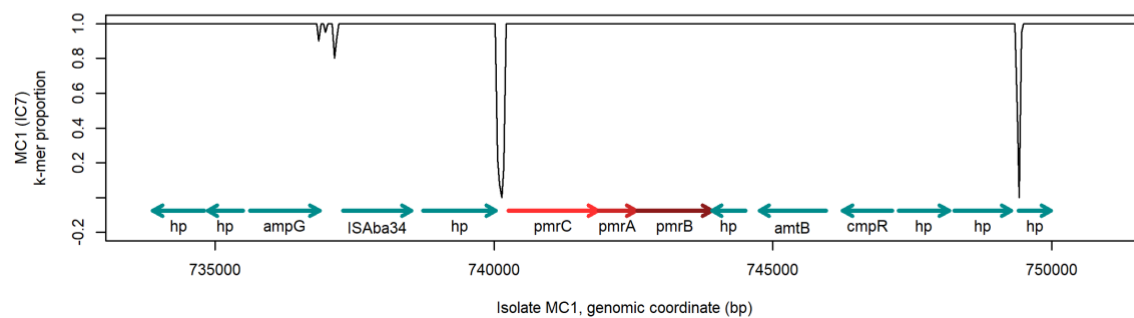

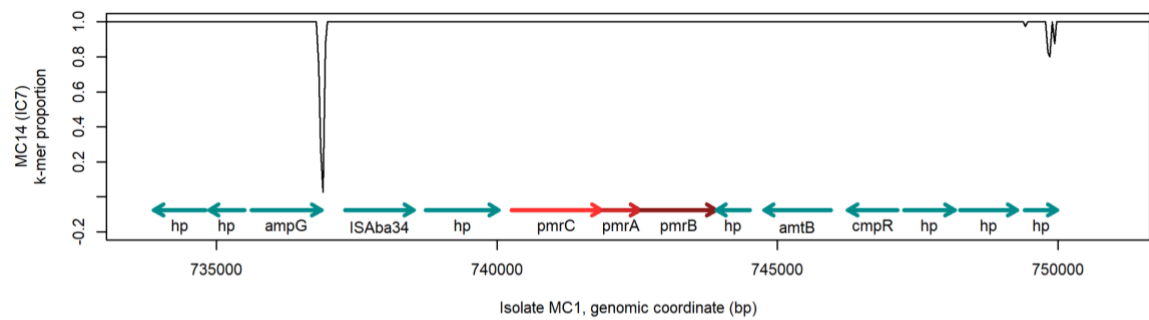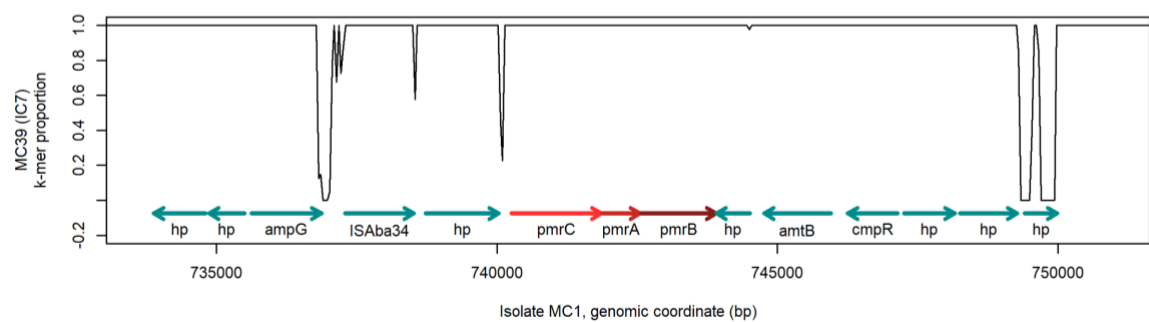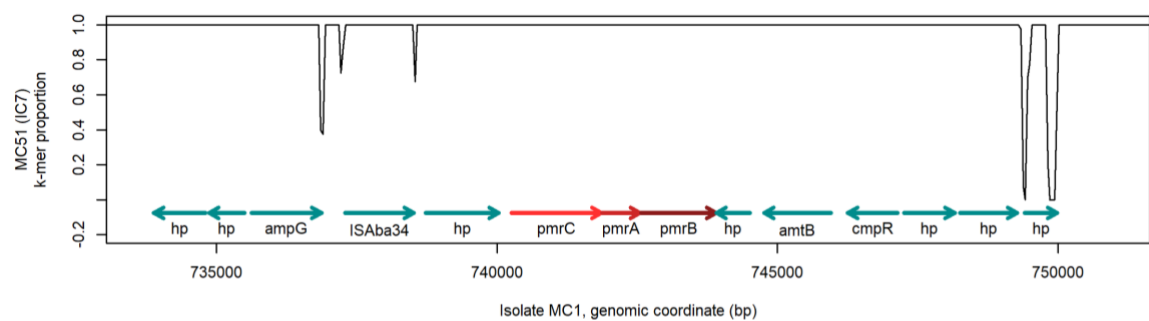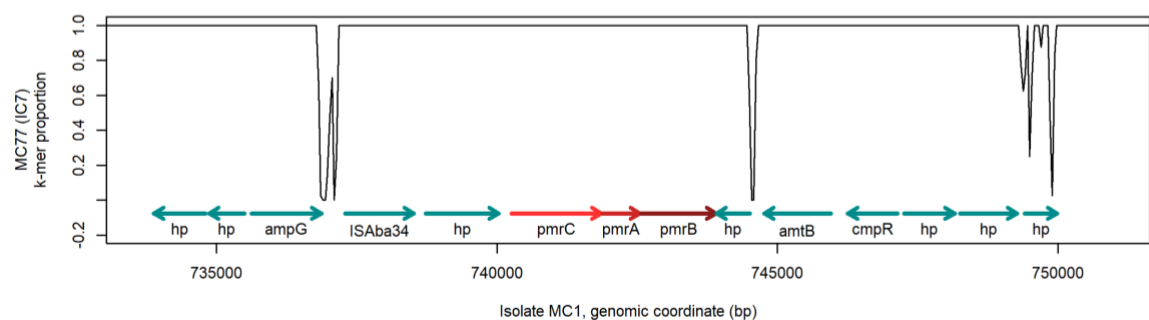

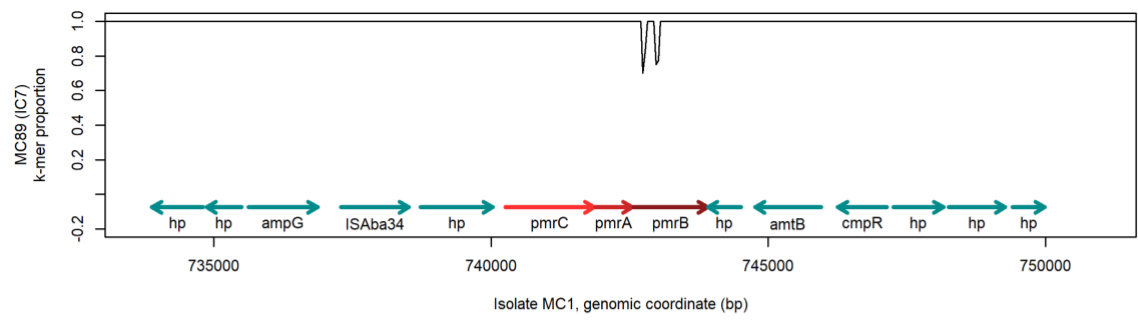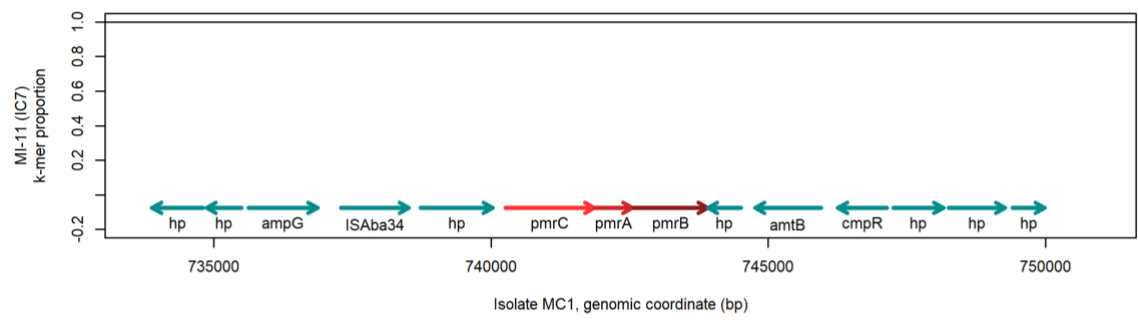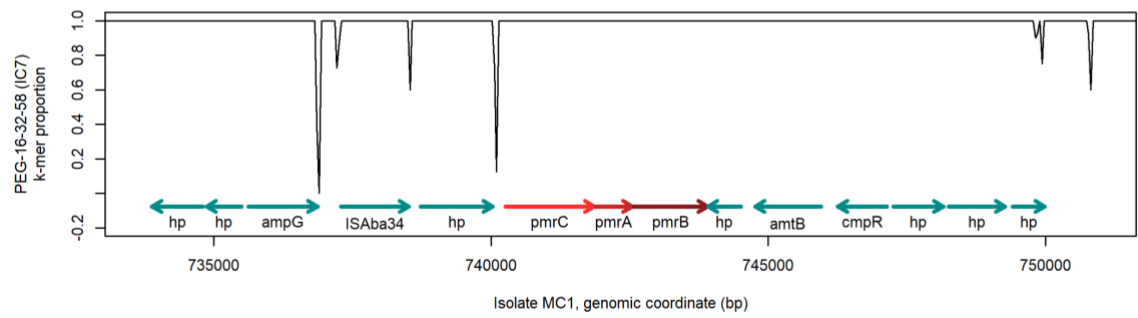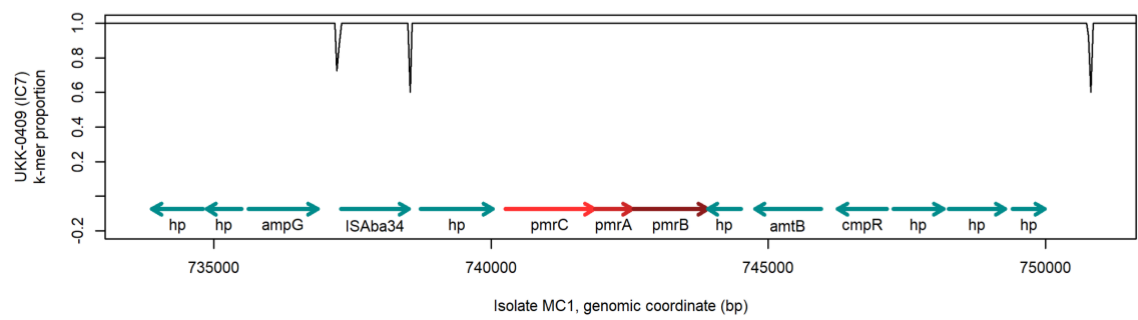

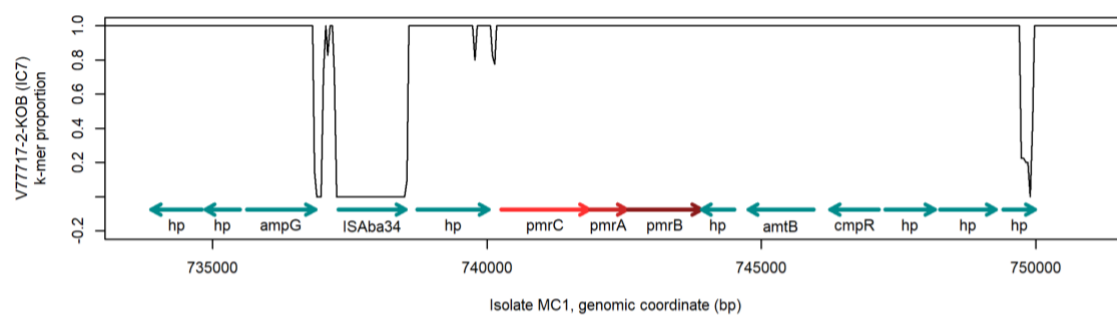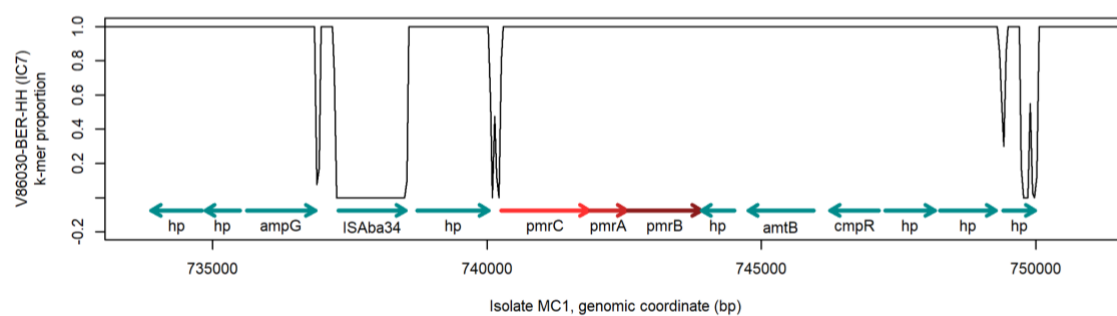

Supplement: FIG S1 [file msphere.00746-21-sf001.pdf]

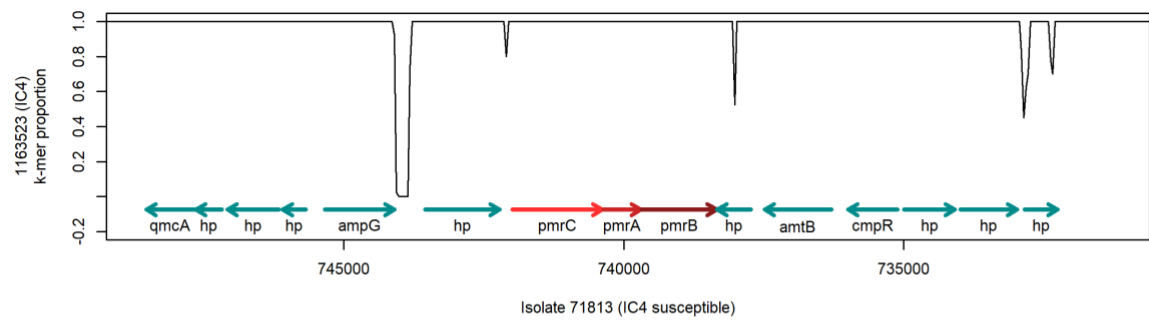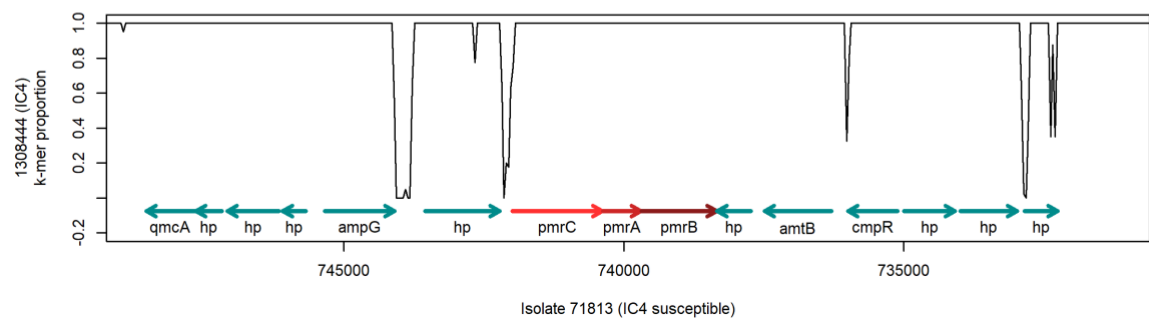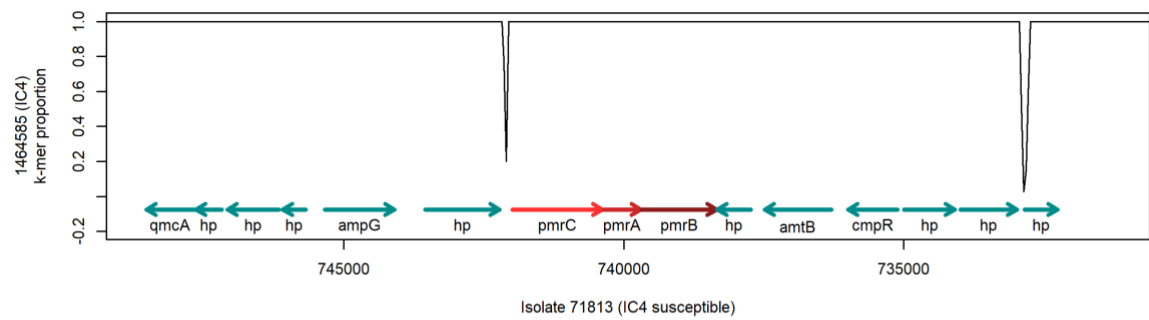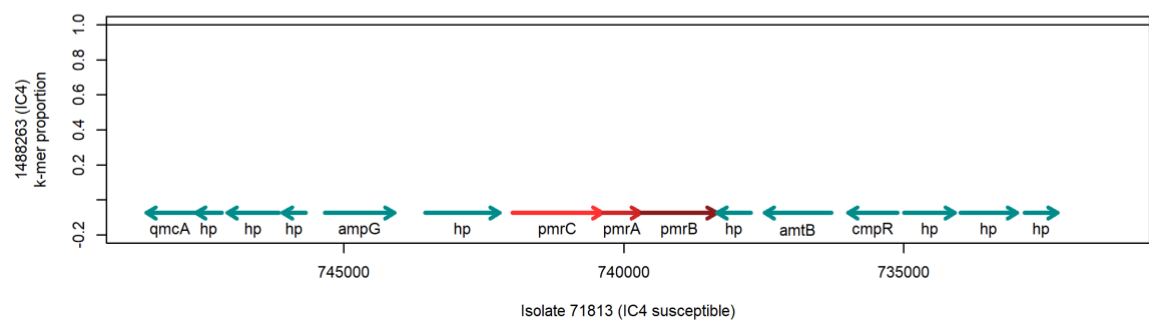

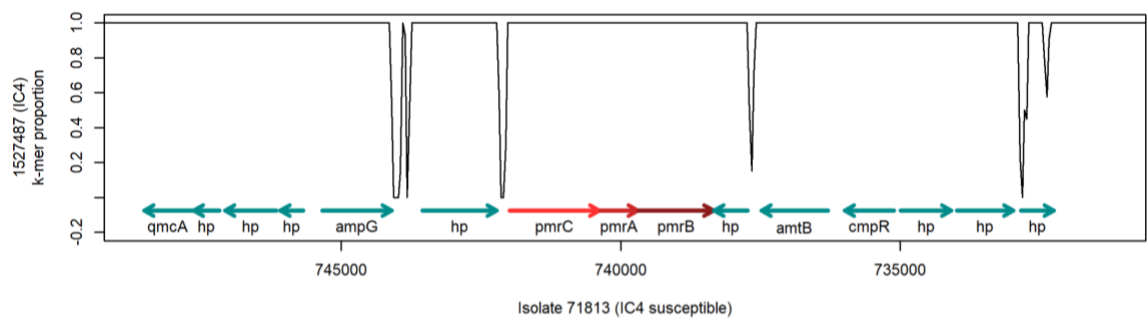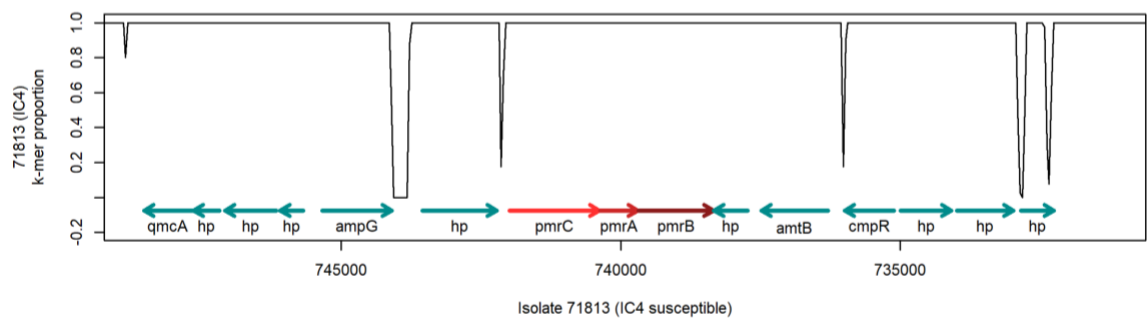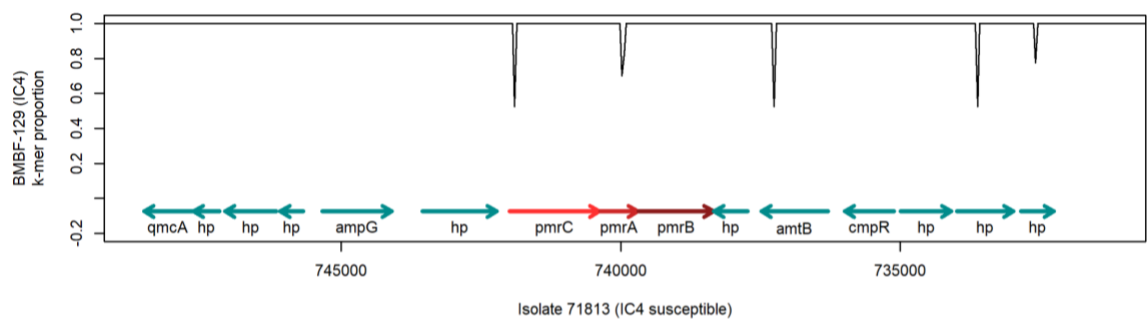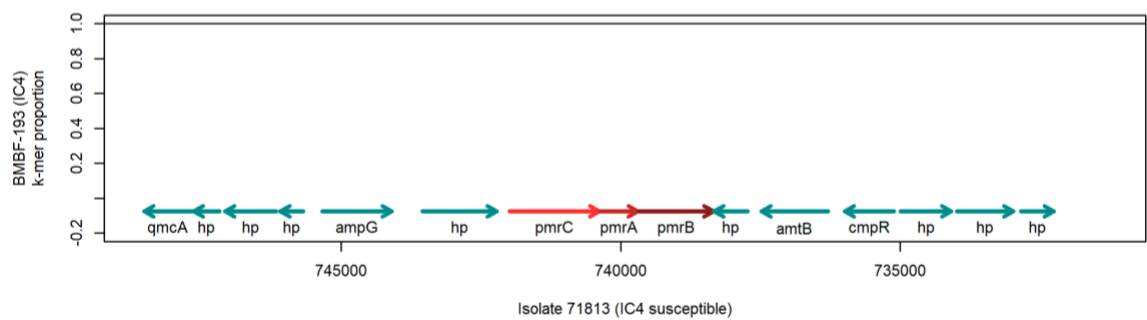

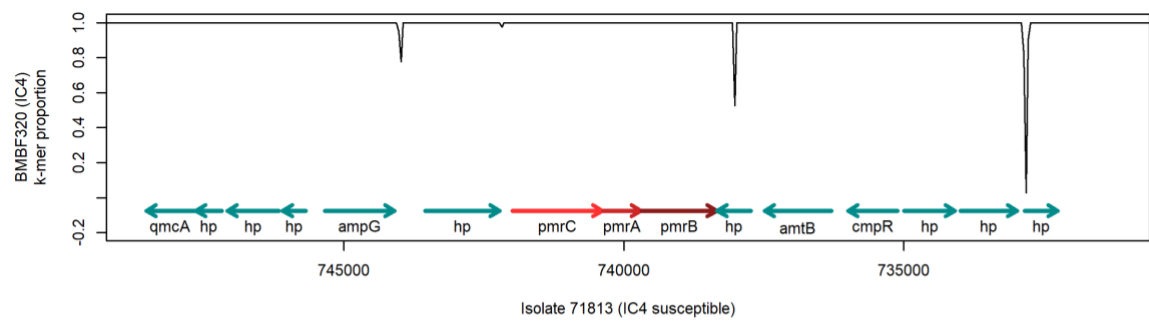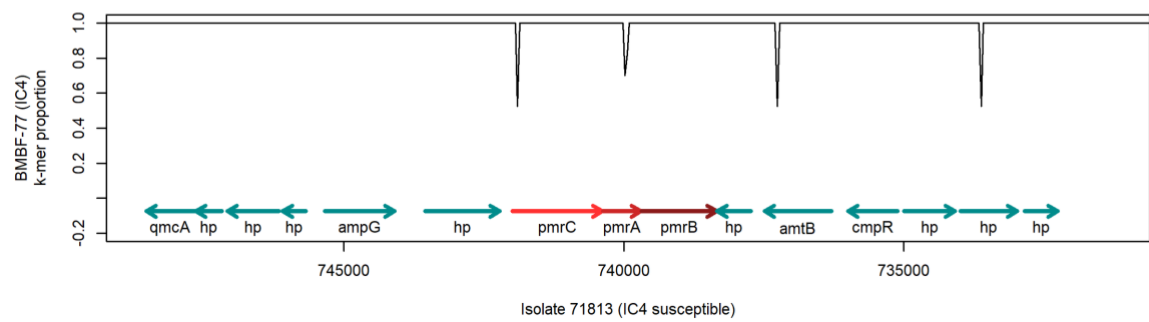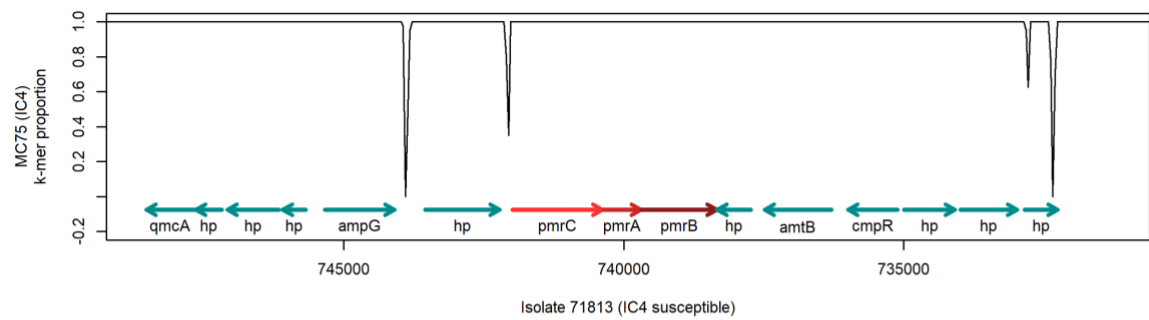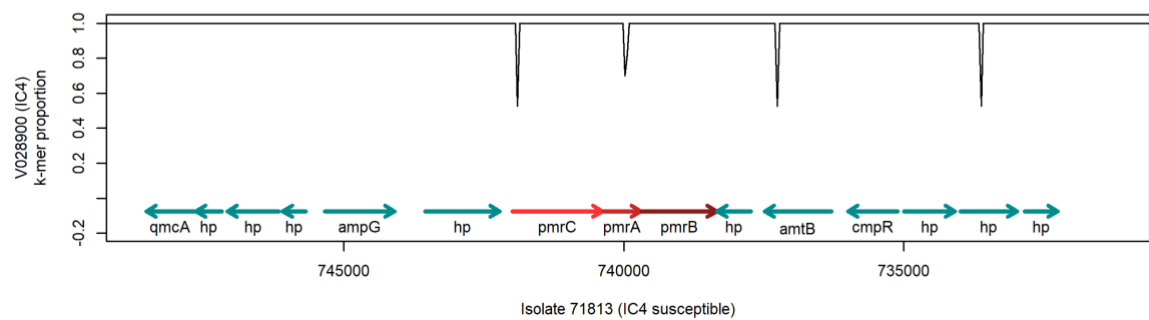

Supplement: FIG S2 [file msphere.00746-21-sf002.pdf]

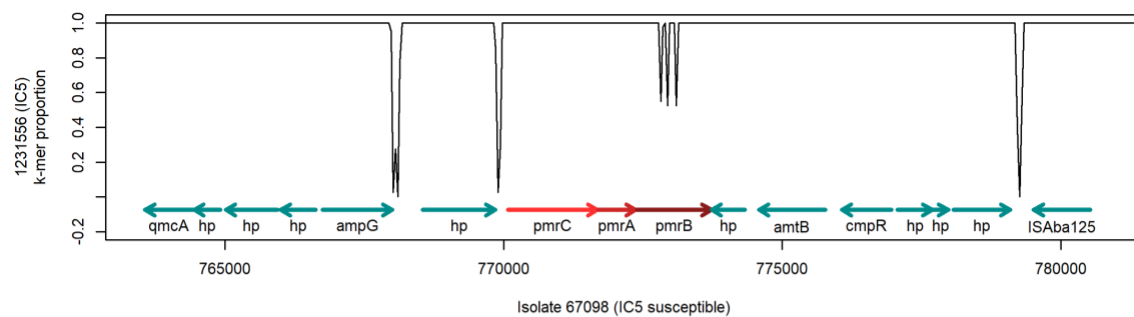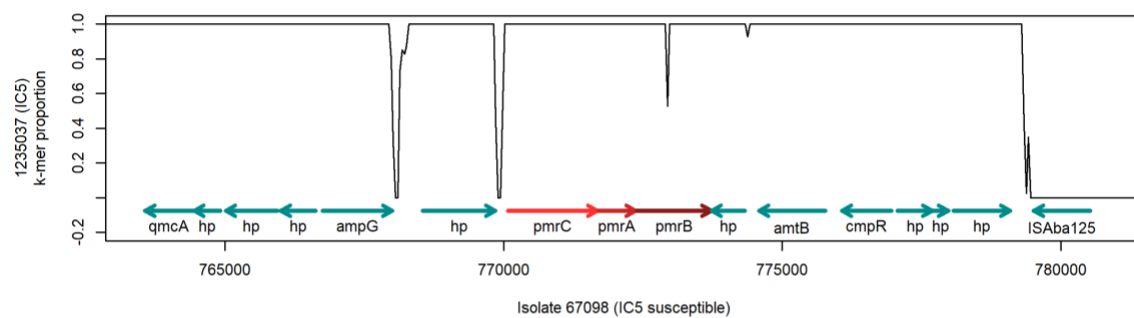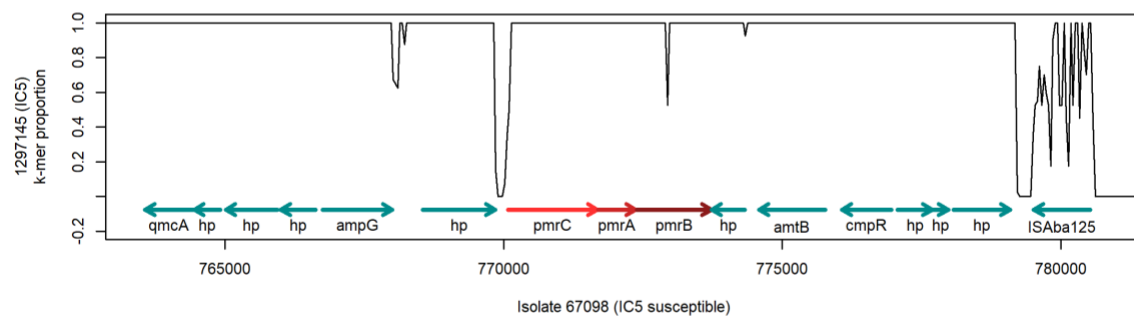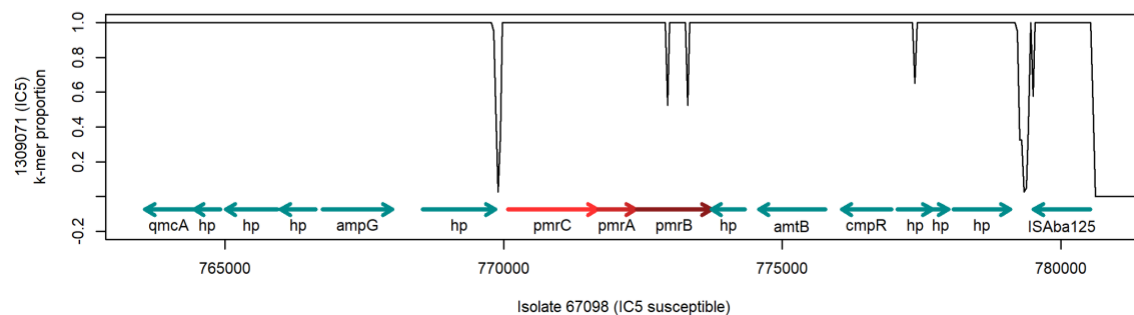

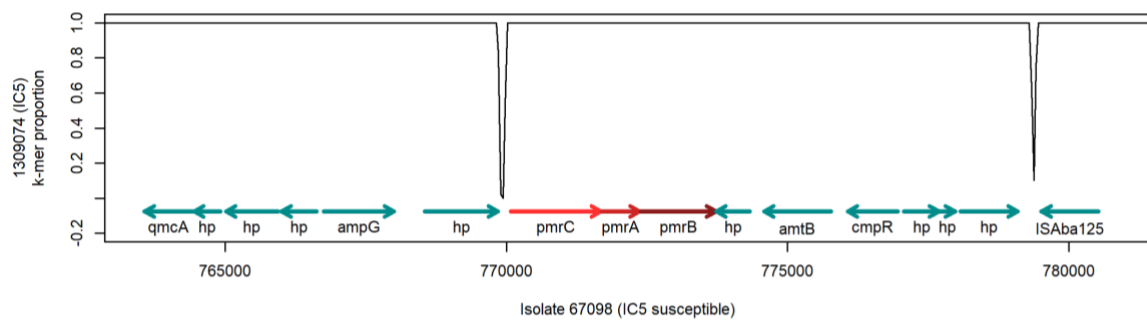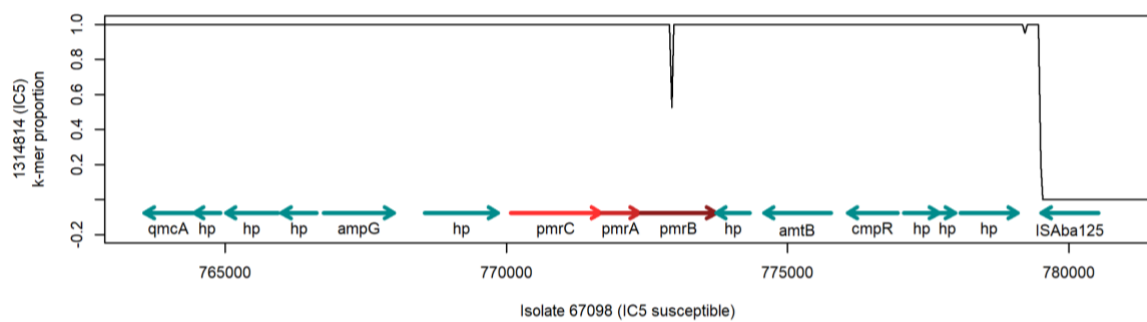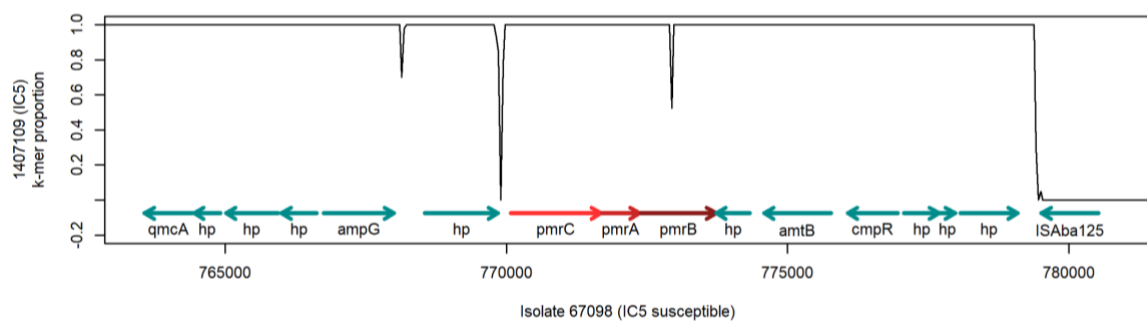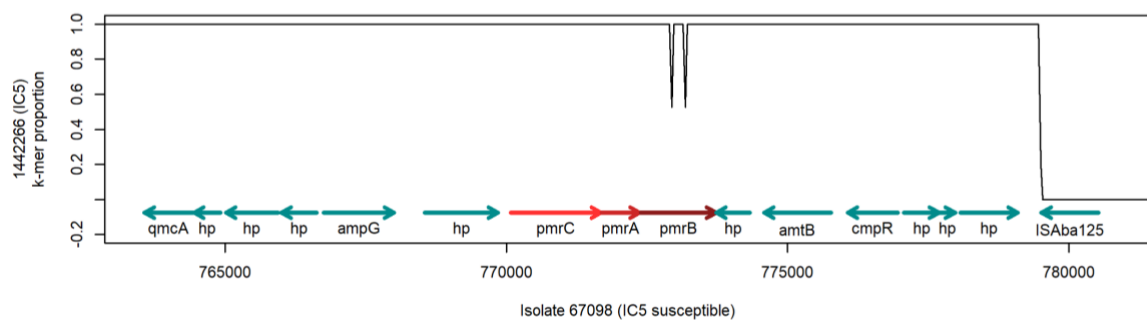

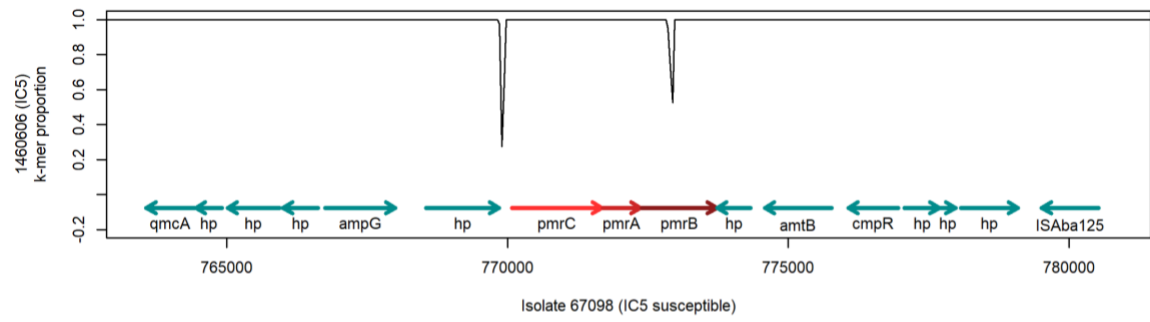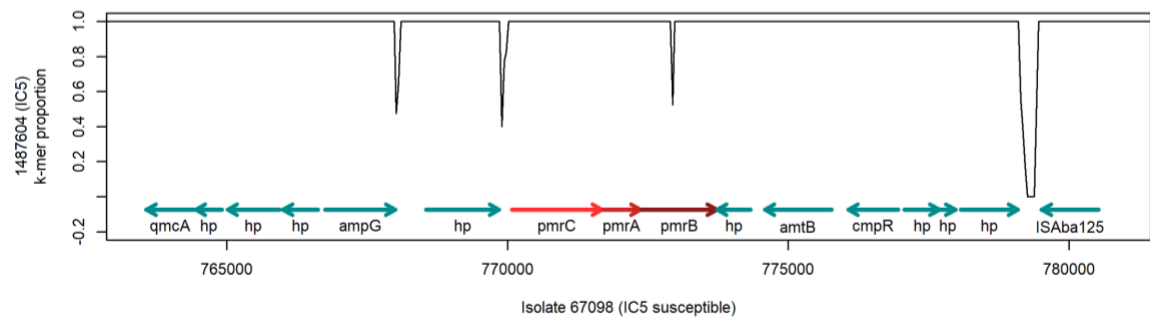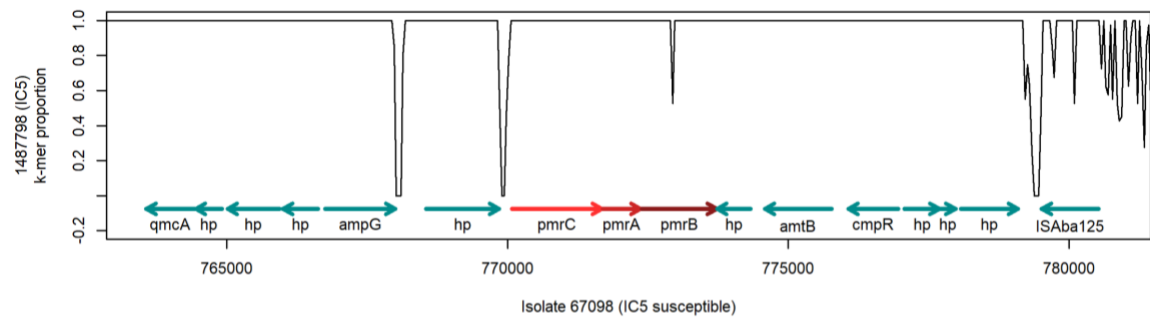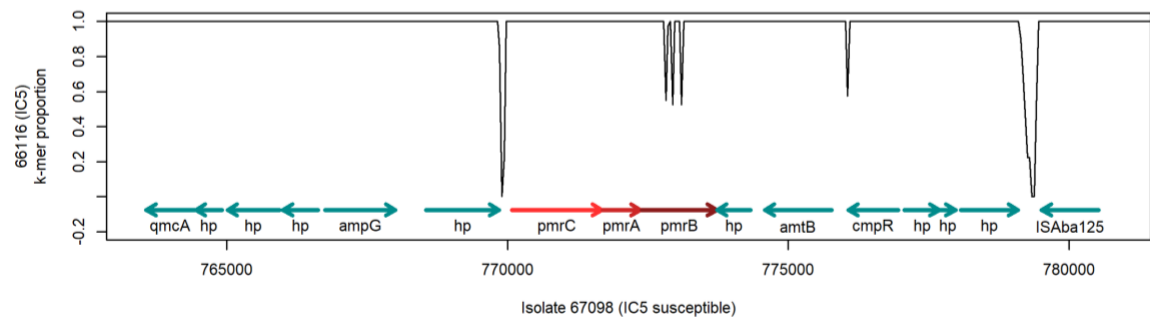

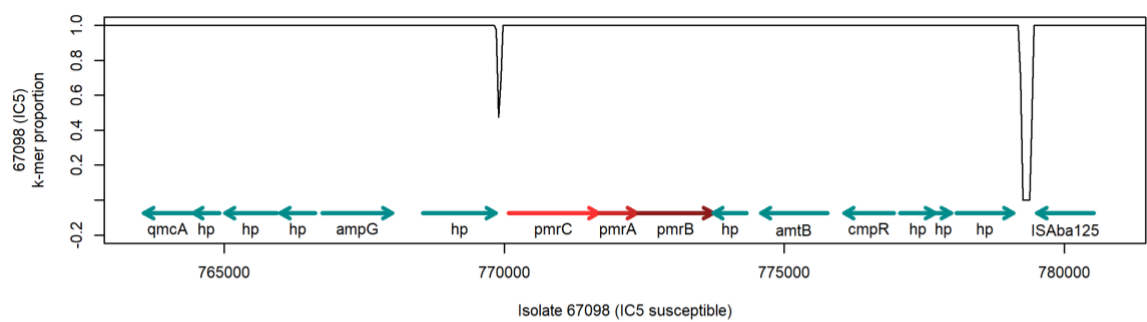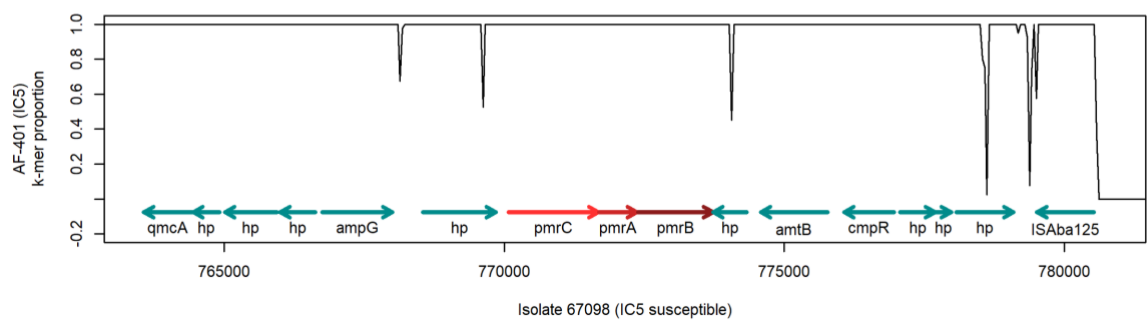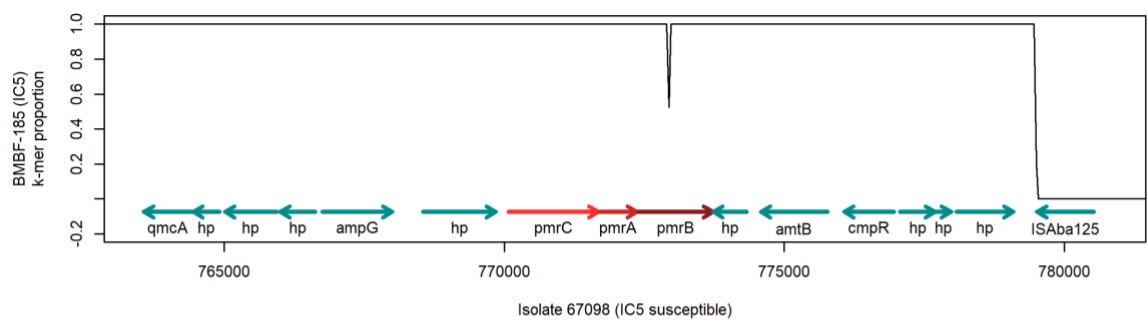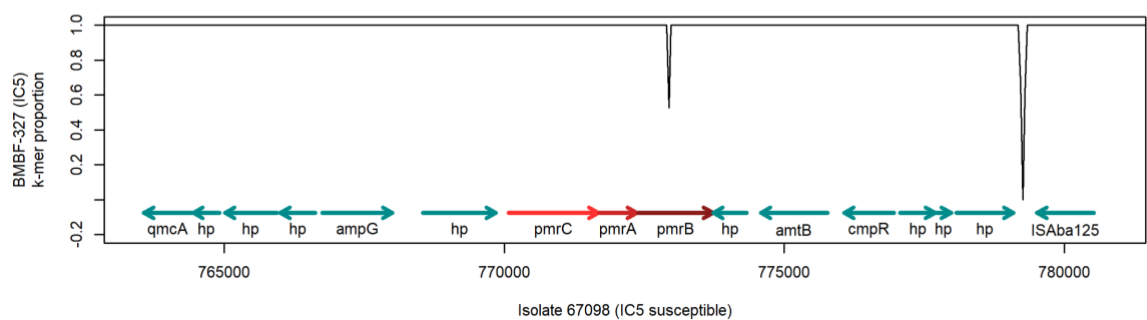

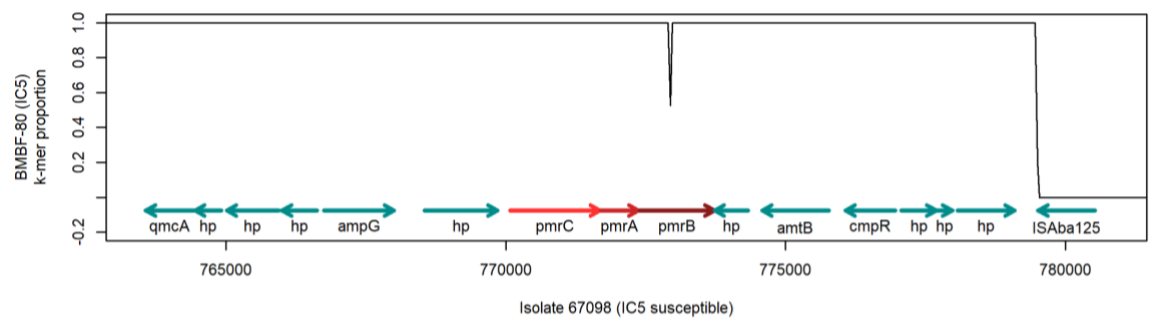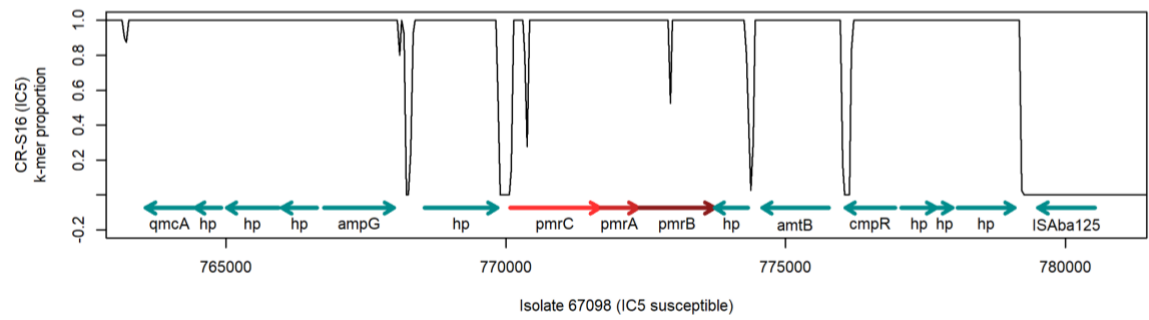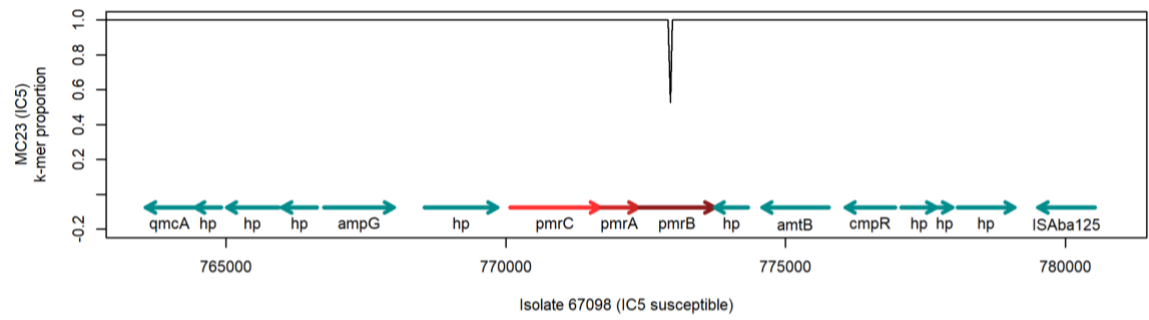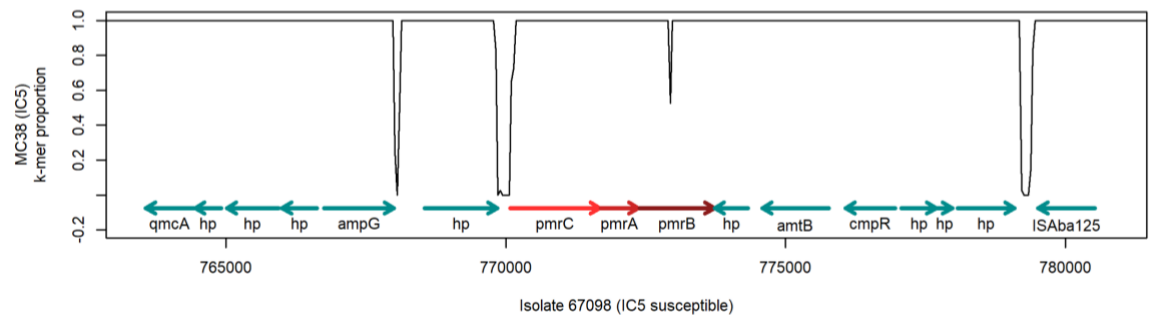

Supplement: FIG S3 [file msphere.00746-21-sf003.pdf]

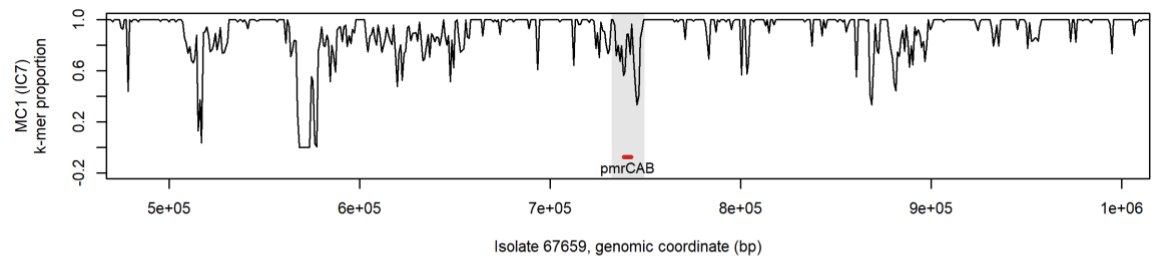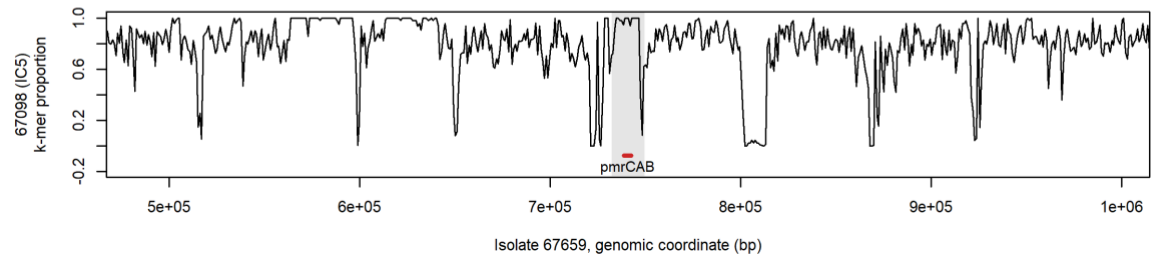

Supplement: FIG S4 [file msphere.00746-21-sf004.pdf]

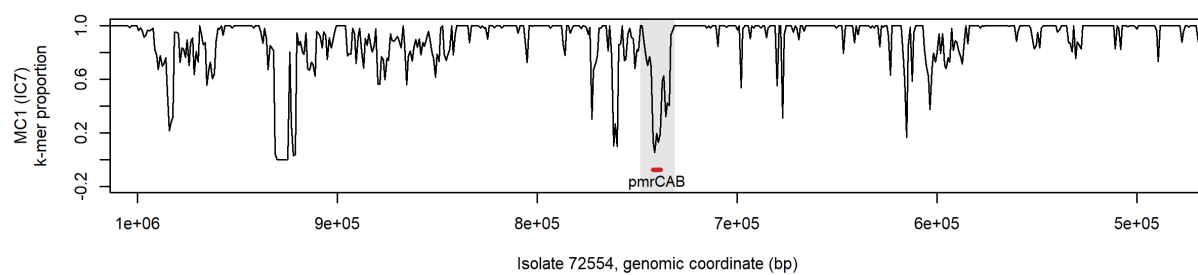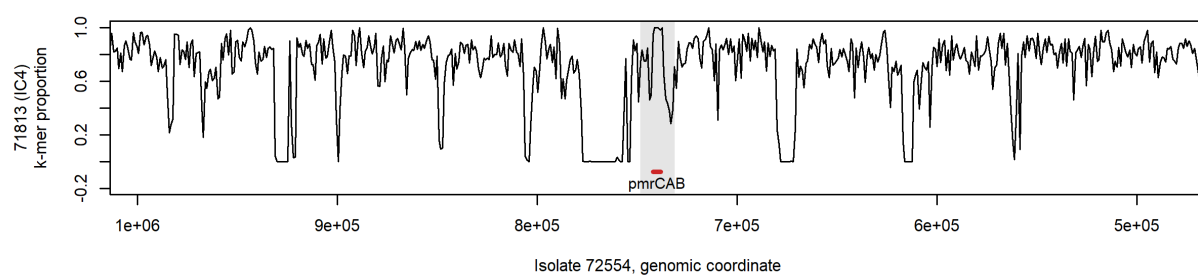

Supplement: FIG S5 [file msphere.00746-21-sf005.pdf]
