# Supplementary material for: pmrCAB Recombination Events among Colistin-Susceptible and -Resistant Acinetobacter baumannii Clinical Isolates Belonging to International Clone 7
Source: mSphere. 2021 Dec 1;6(6):e00746-21. doi: 10.1128/msphere.00746-21 (PMC8636104; doi:10.1128/msphere.00746-21)
Supplement: TABLE S1 [file msphere.00746-21-st001.pdf]

Isolate	Short-reads coverage	Long-reads coverage	Number of contigs[a]	N50[a] (bp)	Number of chromosome-associated contigs[b]	Length of chromosome-associated contigs (bp)	Number of plasmid-associated contigs[b]	Length of plasmid-associated contigs (bp)	Undefined contigs[c]	Genome closed	Inferred localization of *pmrCAB*
67659	116.096	70.689	6	4,174,758	1	4,174,758	3	120,650[d]; 40,936[d]; 16,673[e]	2	yes	chromosome
72554	107.564	42.227	11	2,988,108	2	2,988,108; 1,171,702	3	156,00[d]; 8,970[e]; 7,703[e]	6	yes	chromosome
71813	125.569	311.889	6	3,902,837	2	3,902,837; 34,129	1	14,782	3	yes	chromosome
67098	121.990	129.754	2	4,073,402	1	4,073,402	1	16,673	0	yes	chromosome
MC1	84.566	278.578	3	4,026,212	1	4,026,212	2	184,748; 8,731	0	yes	chromosome

[a] Based on hybrid assembly using Unicycler.

[b] Based on the gene content identified with genome annotation.

[c] All undefined contigs are <5 kbp.

[d] >90% sequence similarity and coverage with the 184 kbp plasmid from MC1.

[e] >99% sequence similarity and coverage with the 16 kbp plasmid from 67098.
